# Some current applications, limitations and future perspectives of lactic acid bacteria as probiotics

**DOI:** 10.1080/16546628.2017.1318034

**Published:** 2017-05-03

**Authors:** Smith Etareri Evivie, Gui-Cheng Huo, John Oamen Igene, Xin Bian

**Affiliations:** ^a^ Key Laboratory of Dairy Sciences, Ministry of Education, College of Food Science, Northeast Agricultural University (NEAU), Harbin, PR China; ^b^ Department of Food Science and Human Nutrition, Faculty of Agriculture, University of Benin, Benin City, Nigeria

**Keywords:** Lactic acid bacteria, probiotics, obesity, type 2 diabetes, food, health

## Abstract

Several mechanism and non-mechanism-based studies supporting the claim that lactic acid bacteria (LAB) strains confer health benefits and play immune-modulatory roles were examined in this review. Probiotic applications of LAB on global burdens such as obesity and type-2 diabetes were discussed as well as the use of yoghurt and ice cream as important vehicles to convey several beneficial LAB strains. Probiotic and symbiotic dairy products may be used in the nearest future to treat a variety of health disorders. Current studies suggest that lactic acid bacteria possess anti-obesity and anti-diabetic propensities on their hosts and thus can play a crucial role in human health care. Research in the rheological and physicochemical properties of ice cream as well as its applications are also on the increase. These applications face certain hurdles including technological (for less developed countries), consumer acceptability of new functional foods may be influenced by culture, ethics or religion. There is need for more studies on the genetic basis for probiotic properties which will give further understanding regarding novel manipulation skills and applicability in nutrition and health sectors. More studies confirming the direct effects of probiotic LABs in lowering the spread of food-borne and other pathogens are also anticipated.

## Introduction

Nutritious foods sustain the complex requirements of the human organism, compensating the limitations of our digestive physiology and anabolic restrictions. Foods are also the perfect media for the growth of microorganisms rendering them inedible and even dangerous for consumption. It has been an everlasting struggle for humans to keep food safe and wholesome [1]. Today, lactic acid bacteria (LAB) species have become an industrially important group of bacteria used for the production of fermented foods such as yoghurt, cheese and butter. They are also crucial microbes featured in many processes used to transform and preserve our foods [2,3]. LABs are widely distributed in the natural world and various species have been used for the production of fermented milk in many countries for thousands of years in the field of food processing [4]. While some act as colonizers on the mucosal surface of our gastrointestinal tracts, others are marketed as probiotics (used in foods to improve nutrition and health) [3]. Probiotics have been defined as ‘live microorganisms (bacteria or yeasts) which when ingested or locally applied in sufficient numbers confer one or more specific demonstrated health benefits for the host’ [5]. This appears to be the most widely accepted definition. The use of probiotics has been proposed in treating other health disorders such as lactose intolerance, food allergy, Crohn’s disease, decreasing the duration of acute infectious diarrhea in infants and children, rheumatoid arthritis and colon cancer [6,7]. For the use of LAB as probiotics, some desirable characteristics such as low cost, maintaining its viability during the processing and storage, facilitation of the application in the products, maintaining viability during upper gastrointestinal passage and resistance to the physicochemical processing must be considered [8]. The most commonly used forms of probiotics are of the genera *Lactobacillus* and *Bifidobacterium* with various strains demonstrated excellent *in vitro* and *in vivo* properties. Although the detailed study of the latter genera is not within the scope of this review, several studies have been duly cited here which also suggest that *Bifidobacterium* sp. can be used for extensive probiotic applications in the nearest future.

In the pharmaceutical industry, LAB strains have also had crucial applications in food formulations via several techniques such as microencapsulation and nanoencapsulation. Microencapsulation technique has been shown to increase survival rates of sensitive microorganisms against a range of unfavorable external conditions in the host [9]. The use of polymer microcapsules as delivery systems has been reported and reviewed in recent literature to have desirable pharmaceutical applications [10]. How flexible or rigid they are plays an important role in their structure–function relationship as well as their scope of application in the food and biomedical industries [11,12].

In recent years, more research interests have focused on the use of probiotics in the treatment of diverse ailments and diseases in view of the findings of various *in vivo* and *in vitro* studies which suggest a strong correlation between these beneficial microbes and the human immune-modulatory responses. It becomes necessary to review some areas where probiotic LAB research have been intensely applied in recent years as well as give meaningful projections in view of future applications. The objective of this review is therefore to give an overview of the main areas of recent applications of LAB as probiotics in improving human health, some of the challenges this research technology still faces and to provide some prospects as to crucial roles they may play in the nearest future.

## Probiotic lab and obesity treatment

Obesity is a serious problem in industrialized and developing countries worldwide. In 2008, more than 1.4 billion adults aged 20 years and above were overweight. Two hundred million of them were men and approximately 300 million were women with the rest being children. Child obesity and being overweight in general has been reported to have increased by 80% from 2000 to 2010 with more than 40 million children under the age of 5 years being overweight. Worldwide level has nearly doubled since 1980 and this calls for concern [13]. As the prevalence of obesity has increased, so also has the prevalence of obesity-related diseases. These include type 2-diabetes, cardiovascular diseases, several types of cancer (endometrial, postmenopausal, breast, kidney and colon cancers), muscle-skeletal disorders, sleep apnea and gall bladder disease [14,15]. Thus, obesity does not only have a devastating effect on health but also a deleterious effect on a nation’s economy. Obesity can be caused by a range of factors including reduction in energy expenditure [16,17] and increases in energy intake [18]. Intestinal environmental factors have been shown to be related to obesity in mice and humans [19,20].

Previous studies have provided some evidence of the anti-obesity potentials of LAB.

It was reported that the consumption of *L. paracasei* ssp paracasei NTU101 or the supernatant of soy milk fermented by NTU101 (SM101) produced anti-obesity effects in HFD-fed rats with body weight, body fat and feed efficiency significantly decreased [21]. The authors also reported that the HFD supplemented diet not only lowered the average radius of adipocytes but also increased the number of small adipocytes, thus demonstrating reduced accumulation of body fats. In addition, Kadoka et al. [22] also reported that the consumption of *L. gasseri* SBT2055 reduced abdominal adiposity, body weight, BMI and weight and hip circumference. Probiotics containing LAB can also be used to improve intestinal barrier functions, thus preventing infection and inflammation. Ouwehand et al. [23] reported that the adhesion of *B. lactis* Bb12 to a mucus model more than doubled following treatment with the GG strain of *L. casei* or *L. bulgaricus*, preventing absorption and colonization by the pathogen on the intestinal epithelial cells. By doing so, LAB may lower the release of LPS from intestinal epithelial cells as well as lower pro-inflammatory cytokine production in adipose tissues. However, in some studies some Lactobacillus and Bifidobacterium strains caused the rodents to gain weight [24]. Nevertheless, with the wide range of fermentable substances that can be used to produce products containing LAB strains, future dietetic probiotics will represent a crucial contribution to research efforts in reducing worldwide obesity [25].

In a recent review by Tsai et al. [25], some mechanisms by which gut microbiota attenuates obesity were elucidated. These include modulation of energy metabolism enzymes, processing of dietary polysaccharides and inducing low-grade inflammation. The modulation of the gut microbiota in the treatment of metabolic syndrome (MS) (caused by obesity and other factors) using three strains of probiotics namely *Lactobacillus paracasei* CNCM I-4270,. *Lactobacillus rhamnosus* CNCM I-3690 and *Bifidobacterium lactis* CNCM I-2494 in HFD-fed mice have been reported in a recent study [15]. They noted that not all the gut bacteria changed by these probiotics were relevant to the probiotic-mediated improvement of MS and also, the strain-specific modulating effects of Lactobacillus and Bifidobactium probiotics on functionally relevant phylotypes were reflected in differential attenuating impacts of these strains on obesity comorbidities. Although all strains tested in this study significantly attenuated HFD-induced weight gain, improved glucose-insulin homeostasis and reduced hepatic steatosis, some studies have suggested that the combination of probiotic strains with complementary effects on gut microbiota and host health will comprehensively improve the different symptoms of MS and thus generate optimal synergistic efficacy. Thus, a study using multiple probiotc-strain formula and to test this formula at different cell densities to determine the optimal dosage becomes imperative [26]. These and other related suggestions have been strongly supported in a recent review by Mekkes et al. [27], who opined that the use of probiotics can be an effective means of treating obesity. Some of the recent studies on the anti-obesity effects of probiotic LABs are as shown in Table 1.

**Table 1.** Some studies on anti-obesity effects of lactic acid bacteria.

Lactic acid bacteria strain	Source	Mechanism of action	Reference
*Lactobacillus plantarum* DK211	Kimchi samples	Prevention of weight gain and body fat accumulation in diet-induced obese rats. Significant decrease in organ weight except in the weight of testis. Significant decrease in total cholesterol, LDL-cholesterol, and triglycerides. Significant decrease in blood glucose levels, and plasma levels of insulin, leptin, and ghrelin.	[28]
*Lactococcus lactis* R704			
*Lactobacillus casei* LcS	Fermented milk drink	Body weight, body mass index, fat mass, leptin and glucose levels were lower and high-density lipoprotein and adiponectin levels were higher in the HFD-LcS and HFD-orlistat groups than in the HFD group. A significant difference in body fat mass was observed between HFD-LcS groups with HFD-orlistat group (19.19 ± 5.76 g vs. 30.19 ± 7.98 g).	[29]
*Lactobacillus paracasei* CNCM I-4270, *Lactobacillus rhamnosus* CNCM I-3690 and *Bifidobacterium animalis* subsp. *lactis* CNCM I-2494	Vegetable and dairy products	Each strain selectively altered a specific subset of key bacteria species that were significantly associated with MS.	[15]
		Partially reversed HFD-induced structural changes in the gut microbiota Significant reduction in TNF-α expression in HFD-fed mice.	
*Bifidobacterium breve* B-3	Healthy infant	Significantly lowering fat mass in human subjects. Improvements in some blood parameters related to liver function and inflammation.	[30]
*Lactobacillus gasseri* SBT2055	Fecal specimen of a healthy adult	LG2055 dose-dependently suppressed lipase activity in the fat emulsion assay. LG2055 dose-dependently increased fat emulsion droplet size.	[31]
*Lactobacillus rhamnosus* GG *Lactobacillus paracasei* TMC0409		Significant reduction in weight, fat accumulation and adipocyte fatty acid synthase (FAS) activity. Significantly increased adipocyte lipolysis. Significant suppression of the adipose tissue gene expression of 11ß-hydroxysteroid dehydrogenase. Stimulating significant up regulation of skeletal muscle and causing significant decrease in the plasma concentration of insulin, malondialdehyde, TNF-α, monocyte, chemodactic protein-1 and C-reactive protein.	[32]
*Streptococcus thermophilus* TMC1543			
*Lactobacillus paracasei* subsp. *paracasei* NTU 101 (W101)	Vegetable product	Decrease in formation of lipid plaques in the aorta, reduced the adipocyte cross-sectional area and diameter, and reduced the levels of CCAAT/enhancer-binding protein β (C/EBPβ), peroxisome proliferator associated receptor γ (PPARγ), and C/EBPα. Suppression of heparin-releasable lipoprotein lipase (HR-LPL) in adipose tissues and inhibited lipid absorption, thereby reducing lipogenesis.	[33]
*Leuconostoc mesenteroides*	Naturally-fermented Kimchi	Significant decrease in triglyceride levels and increased levels of intracellular glycerol and lipid lipolysis. Stimulated reduction in the mRNA levels of PPAR-γ, C/EBP-α, and FAS, which are related to adipogenesis/lipogenesis in 3T3-L1 cells.	[34]
*Lactobacillus plantarum*			
*Lactobacillus plantarum* OLL2712	Fermented milk	Significantly deceased the production of proinflammatory cytokines in vitro. Significantly suppressed proinflammatory cytokines levels in both visceral adipose tissues and the serum of KKAy mice, and reduced serum triglycerides concentration/Alleviation of oxidative stress and adrenaline levels in the serum of KKAy mice.	[35]
*Lactobacillus plantarum* Q180	Fecal samples of healthy adults	Showed a lipase inhibitory activity of 83.61 ± 2.32% and inhibited adipocyte differentiation of 3T3-L1 cells (14.63 ± 1.37%) at a concentration of 100 μg/mL. It also did not produce carcinogenic enzymes such as β-glucuronidase.	[36]
*Lactobacillus plantarum*		Positive correlation with endotoxin levels and lowering of body weight in studied patients.	[37]
*Lactobacillus gasseri* SBT2055	10% fat diet	Significant reduction in body weight and fat tissue mass (epididymal and perirenal/retroperitoneal), with a lowered level of triglyceride content in the liver. DNA microarray analysis showed that LG2055 generally inhibited the up-regulation of pro-inflammatory genes, including CCL2 and CCR2, in the epididymal adipose tissue. LG2055 tended to inhibit lipogenic gene up-regulation, including ACC1, FAS and SREBP1 in the liver. Real-time PCR analysis confirmed the DNA microarray results in part, showing a significant reduction in the mRNA expression of CCL2 in the epididymal adipose tissue, and a downward tendency in FAS mRNA expression in the liver.	[38]
*Lactobacillus paracasei* NTU 101 and *Lactobacillus plantarum* NTU 102	Soy milk fermented products (SM101 and SM102)	Inhibition of 3T3-L1 differentiation and the accumulation of free fatty acids markedly increased in rats. Greater up-regulation and down-regulation of lipolysis and heparin-releasable lipoprotein lipase, respectively, were observed in the 3T3-L1 adipocytes of treated rats.	[21]
*Lactobacillus rhamnosus* GG and *Lactobacillus sakei* NR28	Korean Kimchi	Significant reduction of epididymal fat mass, as well as obesity-related biomarkers like acetyl-CoA carboxylase, fatty acid synthase, and stearoyl-CoA desaturase-1 in the liver	[39]
*Bifidobacterium pseudocatenulatum* SPM 1204	Fecal samples of healthy Koreans (20–30 years old)	Slightly decreased TC, HDL-C, LDL-C, triglyceride, glucose, leptin, AST, ALT, and lipase levels	[40]
*Bifidobacterium longum* SPM 1205		b-glucosidase, b-glucuronidase and tryptophanase activities were decreased by 28, 26 and 10%, respectively.	
*Bifidobacterium longum* SPM 1207			
*Bifidobacterium breve* B-3	Healthy infant	Lowered body weight gain and visceral fat deposits in a dose-dependent pattern. Improving the serum levels of total cholesterol, glucose and insulin	[41]
*Lactobacillus rhamnosus* PL60	Feces and fermented foods grown in 10% skim milk containing 0.01% linoleic acid (LA)	Production of t-10, c-12 conjugated linoleic acid which reduced body weight without affecting energy intake and significant reduction in white adipose tissues (epididymal and perirenal).	[42]

## Probiotic lab and type-2 diabetes treatment

Increase in obesity levels in the world has been predicted to significantly increase the incidence of type-2 diabetes [43]. Type-2 diabetes (T2D) is a group of metabolic disorders, which is characterised by low-grade inflammation, insulin resistance and ß-cell failure that has become increasingly prevalent worldwide [44]. The estimated proportion of diabetes among adults was 8.3% in 2010, among which T2D accounted for at least 90% [45,46]. This proportion is estimated to increase to 9.9% by 2030 [46].

Several studies probing into the antidiabetic effects of LAB in previous years have been reported. Feeding of *Lactobacillus* GG strains in rats showed significant delay in heightened glucose intolerance and hyperglycemic levels [47]. A previous study showed that probiotic dahi containing *Lactobacillus acidophilus* and *Lactobacillus casei* significantly delayed the progression of diabetes in the high-fat diet animals by showing biochemical changes [48]. Also, Yun et al. [49] investigated the effect of *Lactobacillus gasseri* BNR17 on blood glucose levels and body weight in 6 week old, male C57BL/KS/J db/db mice of type-2 diabetes. The authors reported that administering BNR17 doses of 1010 cfu lowered HbA1c values compared to the control group (4.34 ± 0.21% and 4.70 ± o.52% HbA1c levels respectively) but this was not statistically significant. HbA1c is an important factor used to monitor the long-term blood glucose balance because it reflects the number of glucose molecules attached to hemoglobin in the red blood cells. Although the effects slowly diminished at 12 weeks, they indicated that *L. gasseri* BNR17 may have a positive treatment effect on type-2 diabetes.

In a study by Honda et al. [50], it was reported that viable *L. rhamnosus* GG cells significantly inhibited postprandial blood glucose levels in KK-Ay mice when administered with sucrose or starch. The authors also reported that viable GG cells were able to decrease glucose levels *in vitro* and that they possibly improve insulin imbalances. This result was similar to the findings in an earlier study [51]. It was thus suggested that *L. rhamnosus* GG cells decreased postprandial blood glucose through suppression of glucose absorption by decreasing the glucose available for digestion of sucrose and starch in ICR mice. One main conclusion from this study is that the anti-diabetic activity of lactic acid bacteria on KK-Ay mice differed depending on the bacterial strain and whether the bacteria are viable when it arrives in the intestine. Further studies were thus recommended to determine the underlying mechanisms and to identify other such strains of lactic acid bacteria.

Interestingly, in the last decade, several researches have focused on the mammalian host-gut microbiome, suggesting that the gut microflora may have a crucial role to play in controlling the onset of diabetes mellitus. While studies by Owen et al. [52] suggest that breastfeeding in infancy is associated with a reduced risk of type-2 diabetes later in life, Martin et al. [53] stated that breast milk contains a significant amount of LAB necessary for proper development of the infant gut. Based on these, Yun et al. [49] speculated that LAB in breast milk could be one of the important ingredients present for the prevention of diabetes and thus recommended further studies on this. In another study by Panwar et al. [54], some LAB strains (*L. rhamnosus, L. plantarum, L. johnsonii, L. acidophilus* and *L. casei*) were able to stimulate the secretion of gut hormones GLP-1 and GIP which play a key role in modulating insulin secretion and inhibition of DPP IV. This study showed that probiotic LABs may be used as modulators of incretin hormones which can be explored as potential bio-therapeutics against diabetes. Recently, Xu et al. [44] studied the structural modulation of the gut microbiota in the attenuation of type-2 diabetes using a Chinese herbal formula (Gegen Qinlian Decoction – GQD). Their findings showed the gut microbiota could be modified positively and that berberine could be one of the major active ingredients in GQD that modulated the gut microbiota of patients studied. This was further supported by an earlier study by Zhang et al. [55]. However, the authors reported that although it is still unclear whether the changes in gut microbiota by GQD directly contribute to the improvement of glucose homeostasis, the circumstantial evidence does show that gut microbiota might be involved. As controversial as it may sound, probiotic LABs may play increasingly important roles in the development of anti-diabetic medications, foods and food supplements. A summary of some of the recent studies on the anti-diabetic effects of probiotic LABs are as shown in Table 2.

**Table 2.** Some studies on anti-diabetic effects of lactic acid bacteria.

Lactic acid bacteria strain	Mechanism of action	Reference
*Lactobacillus spp*	Trypsin treatment and specific protein-cleavage activity stimulated greater DPP-IV inhibition. α-glucosidase inhibitory activity was also displayed.	[56]
*Lactobacillus plantarum* NCU116	Significantly affecting biological pathways and processes, including metabolism of lipids, lipoproteins, purine, tryptophan, bile secretion, fatty acid biosynthesis, glycolysis and gluconeogenesis.	[57]
*Lactobacillus spp*	Significant DPP-IV inhibitory activity, this varied among the strains studied.	[54]
*Lactobacillus sakei* OK67	Inhibition of HFD-induced body and epididymal fat weight gain, HFD-induced TNF-α and IL-ß expression. Inhibition of NF-kB activation in the colon. Significant down-regulation of HFD-induced expression of PPARy, FAS and TNS-α in adipose tissues.	[58]
*Lactobacillus paracasei* ssp. *paracasei* F19	Increased plasma C-peptide, plasminogen activator inhibitor-1, leptin and serum high-sensitivity C-reactive protein (hsCRP) after overnight fasting in overweight/obese children.	[59]
*Lactobacillus acidophilus* La-5 and *Bifidobacterium animalis* subsp *lactis* BB-12	After 6 weeks, there was a significant decrease in fructosamine levels (−9.91 mmol/L; *p* = 0.04) and hemoglobin A_1_c tended to be lower (−0.67%; *p* = 0.06) in probiotic group. TNF-α and resistin were significantly reduced in probiotic and control groups (−1.5 and −1.3 pg/mL, −.1 and −2.8 ng/mL, respectively). There was a significant difference between groups concerning mean changes of HbA_1c_ (+0.31 for control group *vs* −0.65 for probiotic group; *p* = 0.02), total cholesterol (+0.55 for control group *vs* −0.15 for probiotic group; *p* = 0.04) and LDL-cholesterol (+0.36 for control group *vs* −0.20 for probiotic group *p* = 0.03).	[60]
*Lactobacillus plantarum* OLL2712	Of all LAB strains used in this study, this strain induced the highest levels of IL-10 production in mouse-derived dendritic cells and peritoneal macrophages.	[35]
*Lactobacillus casei* 2 W *Lactobacillus rhamnosus* 27	High α-glucosidase inhibitory activity *in vitro* as well as high anti-diabetic capability.	[61]
*Lactobacillus* strains (Lb1-15)	Some strains (*L. plantarum, L. fermentum, L*. casei and *L. rhamnosus*) had potent and broad spectrum inhibitory activities (up to 89 %; *p* < 0.001; 500 mg/ml wet weight). They could effectively inhibit beta-glucosidase activity (lactase) as well as alpha-glucosidase activities (maltase, sucrase and amylase). Oral gavage with a *L. rhamnosus* extract (1 g/kg) was able to reduce glucose excursions (area under curve; 22 %; *p* < 0.05) in rats during a carbohydrate challenge (starch; 2 g/kg).	[62]
*Lactobacillus rhamnosus* CCFM0528	In an *in vivo* study using high-fat-fed, streptozotocin-induced type-2 diabetic mice, *L. rhamnosus* CCFM0528 significantly decreased fasting and postprandial 2-h blood glucose, glycosylated hemoglobin (HbA1c), endotoxin, tumor necrosis factor-α (TNF-α), interleukin-6 (IL-6), and interleukin-8 (IL-8) levels compared with the control group (*P *< 0.05). The cytokine gene expressions in spleen were regulated, and the islet cells were protected by the probiotic LAB strain	[63]
*Lactobacillus casei* Zhang	*The LAB strain* significantly increased numbers of *Lactobacillus* and *Bifidobacterium* and decreased *Clostridium* in the intestine (*p* < 0.01). Liver glycogen contents were significantly decreased (*p* < 0.05). In therapeutic group, *L. casei* Zhang administration possessed improved glucose tolerance (*p* < 0.05), which were associated with increased osteocalcin level (*p* < 0.01), improved intestinal bile acids secretion (*p* = 0.060), decreased serum MDA levels (*p* < 0.05) and upregulation of LXR-α, PPAR-γ and AdipoR2 gene expression.	[64]
*Lactobacillus reuteri* GMNL-263	High levels of serum glucose, insulin, leptin, C-peptide, glycated hemoglobin, GLP-1, liver injury markers, lipid profile in serum and liver in high-fructose-fed rats were significantly suppressed after Lr263 administration. Feeding of Lr263 reversed the decreased number of bifidobacterium species and lactobacillus species and increased number of clostridium species induced by high fructose treatment. The decreased activities of hepatic antioxidant enzymes in HFD rats were dramatically reversed by Lr263 treatment. Concentrations of IL-6 and TNF-α in adipose tissue were markedly decreased after Lr263 feeding. Decreased levels of PPAR-γ and GLUT4 mRNA after high fructose treatment were significantly enhanced by Lr263 administration. Lr263 consumption normalized the increased lipogenic gene (Srebp-1c, FAS and Elvol6) expressions stimulated by high fructose.	[65]
*Lactobacillus acidophilus*	Fasting plasma glucose (FPG) results showed that consumption of probiotic supplements prevented a rise in FPG (+28.8 ± 8.5 for placebo *vs*. +1.6 ± 6 mg/dl for probiotic group, p = 0.01). The increase of HOMA-IR (homeostasis model of assessment-insulin resistance) in the placebo group was significantly higher than that in the probiotic group (+2.38 *vs*. +0.78, p = 0.03). Mean changes in serum hs-CRP were significantly different between the two groups (−777.57 for the probiotic group *vs*. +878.72 ng/ml for the placebo group, p = 0.02). Probiotic supplementation led to a significant increase in plasma GSH levels compared to placebo (240.63 *vs*. −33.46 µmol/l, p = 0.03).	[66]
*Lactobacillus bulgaricus*		
*Lactobacillus rhamnosus*		
*Lactobacillus casei*		
*Bifidobacterium breve*		
*Bifidobacterium longum*		
*Streptococcus thermophilus*		
*Lactobacillus acidophilus* La5 and *Bifidobacterium lactis* Bb12	Significantly decreased fasting blood glucose and hemoglobin A1c and increased erythrocyte superoxide dismutase and glutathione peroxidase activities and total antioxidant status compared with the control group. Serum malondialdehyde concentration significantly decreased compared with the baseline value in both groups.	[67]
*Lactobacillus plantarum* FSGB	Shown to have effect on body weight and blood glucose/serum parameters when administered at a dose of 0.5 g/kg in obese db/db mice. FSGB improved insulin and glucose tolerance levels. It also enhanced immune activities by increasing immune cell population and glucose transporter 1 (GLUT1) mRNA expression in L6 cells was up-regulated, showing that FSGB may increase glucose activity transport to target cells.	[68]
*Lactobacillus acidophilus* La5	Probiotic yogurt consumption caused a 4.54% decrease in total cholesterol and a 7.45% decrease in LDL-C compared with the control group. Total cholesterol level (HDL-C ratio and LDL-C:HDL-C ratio) significantly decreased in the probiotic group compared to the control	[69]
*Bifidobacterium lactis* Bb12		
*Bifidobacterium animalis* subsp. *lactis* 420	Reversal of the bacterial translocation process from intestine towards tissue by six weeks of treatment with Bifidobacterium animalis subsp. lactis 420, which improves the animals’ overall inflammatory and metabolic status.	[70]

## Probiotic lab and yoghurt starter cultures

While it is well established that a combination of *Streptococcus thermophilus* and *Lactobacillus delbruekii subsp. bulgaricus* have a synergistically role with the traditional yoghurt starter cultures, some studies have shown that this combination can also antagonistic effects on pathogenic microorganisms [71,72]. Aslim et al. [72] investigated the antagonistic effects of *L. bulgaricus* and *S. thermophilus* (production of bacteriocin-like substances) against *H. pylori* in an *in vitro* study. Results of the study showed that supernatant fluids of these strains had significant antibacterial activity against *H. pylori*. However, when the pH was adjusted to 7.0, only 10 strains of *L. bulgaricus* and *S. thermophilus* showed inhibitory activity against *H. pylori*. Previous studies showed that inhibitory activities of LAB are related to acid production (lactic acid predominantly produced) and the low pH attained [73].

Many LAB strains also secrete exopolysaccharides (EPSs) into their extracellular environment which contribute to the cell protection. With respect to food-grade LAB strains such as *L. bulgaricus*, EPSs are of great importance since they enhance the texture of fermented dairy products. They have been studied extensively and even health-promoting properties of LAB have been ascribed to EPS [3]. The surface layers (S-layers) of the cell wall of *Lactobacilli* have also been the subject of great interest in recent years as they are involved in many functions such as adhesion of *lactobacilli* to human intestinal epithelial cells and to extracellular matrix components as well as used as live antigen delivery vehicles [3]. Previous studies by Duboc and Mollet [74] and Ruijssenaars et al. [75] showed LAB-derived EPS may have beneficial physiological effects on health of consumers. These studies claim that EPS due to their increased viscosity in foods may remain for longer time in the gastrointestinal tract and therefore be beneficial to the transient colonization by probiotic bacteria. Broadbent et al. [76] reported that it was unclear whether the structure of some EPS produced by LAB could be involved in phage sensitivity or insensitivity and thus speculated that, in some cases, EPS could be used as a ‘decoy’ for phage absorption. It will be interesting to see further studies carried out in this regard. The production of heteropolysaccharides (HePS) mainly by lactobacillus, lactococci and streptococci has been extensively reported in previous years [77,78]. More than 27 unique EPS gene clusters and 78 glycosyltransferases have been identified on researches on the gene-coding HePS production in *S. thermophilus*. HePS produced by *L. bulgaricus* and *S. thermophilus* can improve the rheological properties, smoothness, creaminess, mouth feel, texture, stability and water retention capacity, thus allowing for non-fat milk to be added in small amounts which is of economic benefit to the producer. Consumers also gain as HePS ensures the production of lowfat versions (a health benefit) and the prevention of gel fracture (also beneficial to the producer) [3].

An earlier report showed that because yoghurt consumption has been shown to confer health benefits which have been linked to the presence of live, non-pathogenic bacteria, the cultures therein can thus be considered ‘probiotic’ [79]. This intensified research interests to further investigate the probiotic effects of yoghurt and possible modifications towards enhancement. Mater et al. [80] showed that *Streptococcus thermophilus* and *Lactobacillus delbrueckii* subsp. *bulgaricus* survived gastrointestinal transit of 13 healthy volunteers consuming yoghurt. The authors reported that of the 39 fecal samples analysed, 32 and 37 fecal samples contained mean values of 6.3 × 104 CFU/g and 7.2 × 104 CFU/g of viable *S. thermophilus* and *L. delbrueckii* subsp. *bulgaricus* respectively. In a comprehensive review, Iyer et al. [81] reported that other possible probiotic attributes of *S. thermophilus* including stimulation of the gut immune system, alleviating the risk of certain cancers, ulcers and inflammation, improve lactose digestion and lower the spread of vaginal and intestinal infections. Some of the recent studies using novel yoghurt starter cultures are summarized in Table 3.

**Table 3.** Some reported probiotic effects of yoghurt cultures in recent years.

Lactic acid bacteria strain in yoghurt	Action	Reference
*Streptococcus salivarius* subsp. *thermophiles*	Improved hyperglycaemia and impaired glucose tolerance with a dose-dependent effect. There was significant decrease in the concentrations of serum total cholesterol (TC) and triacylglycerol (TG)\with a corresponding significant increase in the concentrations of high-density lipoprotein cholesterol (HDL-C) and insulin. Hypertrophy of liver and kidney were normalized and pancreas islet was restored.	[82]
(GABA-rich yogurt)		
*Lactobacillus acidophilus* ATCC 4356 and *Lactobacillus paracasei* ATCC BAA52	Pineapple peel powder (PPP) supplementation at 1% remarkably reduced fermentation time of milk co-fermented with probiotic organisms. Syneresis level in probiotic yogurt with PPP (1.16% at day 1) was comparable with the prebiotic-inulin and increased during storage. Firmness and storage modulus in both plain and probiotic yogurts were, however, lowered significantly with PPP addition.	[83]
*Lactobacillus acidophilus* La5 and *Bifidobacterium lactis* Bb12	There was no significant change in blood pressure, heart rate or serum lipid concentrations (P > 0.05). No significant changes in blood pressure or concentrations of total cholesterol LDLC, HDLC or triglycerides (P > 0.05) were recorded.	[84]
*Streptococcus thermophilus, Lactobacillus bulgaricus, Bifidobacterium lactis* Bb12 and *Lactobacillus acidophilus* LA-5	Significant reduction in weight, body mass index and serum levels of fasting insulin in 72 subjects. No significant changes were observed in waist circumference, homeostasis model assessment insulin resistance, serum leptin, adiponectin and leptin to adiponectin ratio in both probiotic yoghurt and control.	[85]
*Bifidobacterium animalis* BI-07	Probiotic yoghurt had higher phenolic content (P < 0.05) which suggested higher phytochemical levels. High initial cell count (approx. 99 log CFU/g) and a 98% probiotic survival rate during the 90 days frozen storage period.	[86]
*Lactobacillus acidophilus* and *Bifidobacterium lactis*	The changes were similar among the three groups in terms of decrease in plasma protein carbonyl levels. Although there was no significant change among the three groups significant within-group decreases in plasma iso prostaglandin were observed in the probiotic supplements group (111.9 ± 85.4 vs. 88.0 ± 71.0 pg/mL, *P* = 0.003) and in the probiotic yogurt group (116.3 ± 93.0 vs. 92.0 ± 66.0 pg/mL, *P* = 0.02).	[87]
Multispecies probiotic capsule contained *Actobacillus casei, L. acidophilus, Lactobacillus rhamnosus, Lactobacillus bulgaricus, Bifidobacterium breve, Bifidobacterium longum* and *Streptococcus thermophilus*		
*Bifidobacterium animalis*	Supplemented yoghurt mixes showed greater buffering capacities than non-supplemented yoghurt mixes. After 28 days of storage, *L. bulgaricus* and *L. acidophilus* counts were greater compared to the non-supplemented yoghurts. *Streptococcus thermophilus* and *Bifidobacterium animalis* counts were not affected by supplementation.	[88]
*Lactobacillus acidophilus*		
*Lactobacillus bulgaricus*		
*Streptococcus thermophilus*		
*Bifidobacterium lactis* Bb-12	72 children commenced and 70 children (36 placebo and 34 probiotic) completed the trial. There were no incidents of severe diarrhoea (stool consistency ≥6, ≥3 stools/day for ≥2 consecutive days) in the probiotic group and six in the placebo group (Fisher’s exact p = 0.025). There was also only one episode of minor diarrhoea (stool consistency ≥5, ≥2 stools/day for ≥2 days in the probiotic group compared to 21 in the placebo group (Fisher’s exact p < 0.001). The probiotic group reported fewer adverse events (one had abdominal pain, one vomited and one had headache) than the placebo group (six had abdominal pain, four had loss of appetite and one had nausea).	[89]
*Lactobacillus acidophilus* La-5		
*Lactobacillus rhamnosus* GG (LGG)		
Not specified	Statistically significant differences were observed between both study and control groups regarding to frequency, degree of severity and duration of antibiotic associated diarrhea (AAD).	[90]
*Lactobacillus acidophilus* LA-5, *Bifidobacterium lactis* Bb12, and *L. rhamnosus* (LGG).	Consumption of probiotic milk in pregnancy was associated with a slightly reduced risk of atopic eczema at 6 months and of rhinoconjuctivitis between 18 and 36 months, compared with no consumption during pregnancy. Maternal history of allergic disease did not notably influence the associations. Probiotic milk consumption was not associated with asthma at 36 months.	[91]
*Lactobacillus fermentum*	Body fat mass was reduced in all treatments, with the greatest reduction from LA consumption. Bacterial distribution of gut microflora determined a significant reduction in the abundance of Clostridial cluster IV from LA consumption and significant increases in the abundance of Lactobacillus in both LF and LA treatments.	[92]
*Lactobacillus amylovorus*		
*Bifidobacterium sp*	A significant difference was observed between intervention groups of PI (probiotic yoghurt) and PC (plain yoghurt) with the healthy group (p < 0.05). After the intervention, serum levels of IL-1*β*, TNF-*α* and CRP were significantly decreased in PI group compared to their baseline values and intervention groups. The serum levels of IL-6 and IL-10 increased significantly after the intervention compared to baseline values and PC levels (all p-values < 0.05).	[93]
*Lactobacillus sp*		
*Streptococcus thermophilus, Lactobacillus bulgaricus, Bifidobacterium animalis* and *Lactobacillus acidophilus*	After 9 weeks of consumption, significant difference in serum insulin levels and HOMA-IR score, were found between probiotic and conventional yogurts (changes from baseline in serum insulin levels: +1.2 ± 1.2 *vs*. +5.0 ± 1.1 μIU/ml, respectively, P = 0.02; and in HOMA-IR score: −0.2 ± 0.3 *vs*. 0.7 ± 0.2, respectively, P = 0.01).	[94]
*Lactobacillus reuteri* NCIMB 30,242	Over the intervention period, subjects consuming yoghurts containing microencapsulated L. reuteri NCIMB 30242 attained significant reductions in LDL-cholesterol (LDL-C) of 8 · 92% (P ¼ 0 · 016), total cholesterol (TC) of 4 · 81%	[95]
	(P ¼ 0 · 031) and non-HDL-cholesterol (HDL-C) of 6 · 01% (P ¼ 0 · 029) over placebo, and a significant absolute change in apoB-100 of 20 · 19 mmol/l (P ¼ 0 · 049). Serum concentrations of TAG and HDL-C were unchanged over the course of the study.	
*Lactobacillus bulgaricus, Streptococcus thermophilus, probiotic lactobacillus and Bifidobacterium lactis*	Ten patients in probiotic group and nine subjects in clindamycin group had symptom recurrence (*p *> 0.05). 132 patients in probiotic group and 105 subjects in clindamycin group had pH decrease (*p *< 0.0001). 140 patients in probiotic group and 141 subjects in clindamycin group had complete symptomatic cure (*p *> 0.05). 12 patients in probiotic group and seven subjects in clindamycin group had preterm birth. Nine women in probiotic group and five subjects in clindamycin group had PROM (*p *> 0.05).	[96]
*Lactobacillus acidophilus* and *Bifidobacterium lactis*	Total cholesterol and LDL-cholesterol concentrations decreased by 4.54 and 7.45% in the intervention group (type-2 diabetic volunteers), respectively, as compared with the control values (*P *= 0.008 and *P *= 0.004, respectively).	[97]
*Lactobacillus acidophilus* La5and *Bifidobacterium animalis* BB12	Significant decrease in serum hs-CRP level in pregnant women but no effect on TNF-α.	[98]
Traditional starter culture + *Bifidobacterium lactis* Bb12	Compared to the basal sample, faecal IgA increased during probiotic feeding (P = 0.0184) and returned to normal after cessation of probiotic yoghurt intake.	[99]
*Lactobacillus acidophilus* LA-5, *Lactobacillus bulgaricus, Bifidobacterium bifidum* Bb-12, *Lactobacillus casei* LC-01 and *Streptococcus thermophilus* Th-4 4 in the ratio 4:4:1:1 and a capsule of probiotic mix containing *L. bulgaricus, Lactobacillus rhamnosus, Bifidobacterium infantis* and *Bifidobacterium longum* in the ratio of 1:1:1:1	A. indica-yogurt pH was lower whereas total titratable acid (TTA) was higher than plain-yogurt during storage. A. indica yogurt had highest TPC (74.9 ± 5.1 lgGAE/ml; p < 0.05) on day 28 and DPPH inhibition (53.1 ± 5.0%; p < 0.05) on day 14 compared to plain yogurt (29.6 ± 1.1 lgGAE/ml and 35.9 ± 5.2%, respectively). A. indica yogurt water extract increased the inhibition to maximal values for a-glucosidase and ACE on day 14 of storage (15.9 ± 10.1% and 79.70 ± 11.2%, respectively) and for a-amylase on day 21 of storage (54.8 ± 3.2%). A. indica yogurt has higher TPC, antioxidant activities and enzymes inhibitory effects than plain-yogurt.	[100]

## Probiotic lab in ice cream

Ice cream is defined as a frozen dairy food made by freezing a pasteurized mix with agitation to incorporate air and ensure uniformity of consistency. The mix typically consists of milk products, sugar, dextrose, corn syrup (CS), water, egg or egg products, non-harmful flavourings, stabilizer and emulsifier. Its nutrient composition and energy value is typically a function of the food value of the products from which it is made [101]. Regardless of the mix, ice creams are generally excellent sources of food energy and an ideal substrate for the proliferation of probiotics [102,103].

The use of ice cream in conveying probiotic organisms into the human body has been reported to have more advantages than other fermented dairy products, as its pH is close to neutral which is favourable for the survival and metabolic activity of probiotic bacteria [104]. However, freezing and thawing processes have deleterious effects on probiotics ranging from interruption of metabolic activities and death of cells [105]. Studies geared at limiting these adverse effects have been reported. Leandro et al. [106] studied the survivability of *Lactobacillus delbrueckii* subsp. *bulgaricus* UFV H2b20 in three ice-cream formulations (low-fat, fat-free and high-fat) stored at −16°C for 40 days. They reported that although the survival of this LAB was not significantly affected (P > 0.05) in the three formulations, *Lactobacillus delbrueckii* subsp. *bulgaricus* UFV H2b20 survivability was not lowered by bile acids and salts. In addition, lowering fat content of the formulations did not affect viability count, thus suggesting that probiotic LABs can be incorporated in ice-cream production and the processing parameters can be manipulated for different categories of consumers.

In a recent study using fermented and non-fermented probiotic ice cream containing *Bifidobacterium bifidum* (Bb-12), Aboulfazli et al. [107] reported that fermentation increased the apparent viscosity, hysteresis, particle size and freezable water, all of which are vital parameters in producing desirable frozen desserts. However, the authors also reported that fermentation decreased the melting rate and total acceptability of ice creams. In another study involving the replacement of cows’ milk with soy or coconut milk in ice-cream samples fermented with *Lactobacillus acidophilus* (La-5) and *Bifidobacterium bifidum* (Bb-12), Fatmeh et al. [108] further reported that when cows’ milk was replaced with soy or coconut milk, the probiotic growth (p < 0.05) of Bb-12 (1.2 log10 cfu/g) in fermented ice cream compared to cows’ milk ice cream (0.84 log10 cfu/g). This study also showed that La-5 increased (p < 0.05) by 1.29 log10 in fermented soy milk ice cream compared to cows’ milk ice cream (1.09 log10 cfu/g). They thus concluded that both soy and coconut milk ice creams provide a richer growth medium of amino acids and sugar content (particularly lactose and sucrose) for Bb-12 and La-5. Findings from similar studies on the rheological and physicochemical properties as well as the applications have attracted research interests over the years [109–114].

The use of synbiotics (probiotics and prebiotics) in ice-cream processing may also open new frontiers in its synergistic use. Using a combination of probiotic *lactobacillus acidophilus* La-5 and fructooligosaccharide (FOS) in a yoghurt–ice cream combination, Ahmadi et al. [115] in a recent study showed that alginate-encapsulated microbeads protected probiotic bacteria cells from injury during the freezing process and at frozen storage. FOS incorporation on the other hand (8%) significantly increased overrun of prepared treatments and reduced their firmness. Microencapsulation is highlighted here as a useful technology in enhancing the applicability of ‘probiotic’ ice cream. The phenolic content and antioxidant properties of ice cream were also shown to significantly improve (*P *< 0.05) due to the inclusion of *L. casei* Shirota. These workers showed that the bacteria did not only survive in high amounts (7.21 log cfu/ml) for the 60-day storage period but that all ice-cream samples had generally acceptable sensory scores [116].

## Some current challenges in lab application as probiotics

The application of probiotic LABs has been mitigated by certain challenges, some of which will be highlighted in this review. Although probiotics have many health benefits, these claims can only be asserted if a high number of viable cells reach the small intestine. Many probiotic bacteria have been shown to die in food products after exposure to low pH after fermentation, oxygen during refrigeration distribution and storage of products as well as the acidic conditions in the human stomach [117–119]. The viability and survival of probiotic bacteria are strain-specific, thus microencapsulation techniques such as solvent evaporation have been successfully applied to protect the bacterial cells from damages caused by external environment. In a preliminary microencapsulation study, Evivie [117] showed that more viable *L. plantarum* cells were obtained than *B. breve* cells. There are also challenges with the sensory acceptance of probiotic foods. Some studies have reported the possibility of obtaining similar, or even better, performance with probiotic products as compared to conventional products such as: functional yogurt supplemented with *L. reuteri* RC-14 and *L. rhamnosus* GR-1 [120], chocolate mousse with added inulin and *L. paracasei* [121], curdled milk with inulin, and *L. acidophilus* [122], and milk fermented with *B. animalis* and *L. acidophilus* La-5, and supplemented with inulin [123]. Other challenges as reported by Champagne et al. [124] and Antoine [125] include inoculation, assessment the viable counts of the probiotic strains particularly when multiple probiotic strains are added and when there are also starter cultures added maintaining of probiotics, diversity and origin of probiotics, probiotic survival and being active, dealing with endogenous microbiota and proving health benefits.

Finally, detailed studies of the physiology of LABs regarding they interact with other microbes and as they interact with foods are still lacking. These include a series of mathematical models and technologies investigating LAB bioinformatics, which can be used to reasonably predict responses of LABs in certain food systems as well as explore further applications. Thankfully, research into omics and other analytical technologies is gradually making the picture less hazy but there is still much to be done [126]. Landete [127] recently opined that while genetic engineering of LABs can have several positive effects on the food and pharmaceutical industries, it could be limited by legal issues surrounding the use of the technology, which is still controversial in some quarters. This has, in part, contributed in less studies investigating the use of GRAS recombinant LAB strains that lower the onset of obesity and T2D biomarkers. It is hoped that when some of these hurdles are addressed, more biotechnological and related applications should be in the horizon.

## Some highlights on probiotic lab and the future prospects

There can be no sense of global security without first ensuring food security. This includes, among other factors, the provision of safe and nutritious food for the globe’s teeming population. Given the enormous opportunities that exist in the use of LABs as probiotics, the future is indeed promising. One area, for example, that is currently being tapped into is investigating probiotic propensities through complete genome sequencing technology. This among other impacts will increase the functionality of probiotic LABs and data thereof can be used as a good basis to further manipulate LAB genes [8]. More research into their use as functional food ingredients is currently underway and is expected to increase in the nearest future. There is already growing research into the attenuating effects of probiotic LABs on breast cancer cells and the likes, thus further bridging the gap between the food, health and medicine sectors of the world [128]. It must, however, be stressed that improving food safety is not a probiotic characteristic of lactic acid bacteria (LAB).

In the last decade, more studies investigating the molecular basis for the potential probiotic properties of prospective LAB strains and their products have emerged, radically improving our understanding of their biology [3,129–131]. These reports have formed and can still form the basis for meaningful research *in vitro* and *in vivo* studies which will be of paramount importance to experts in the food, biomedical and pharmaceutical industries. Preliminary findings from ongoing research at the Key Laboratory of Dairy Science (KLDS) of the Northeast Agricultural University (NEAU), China, have shown that a recently sequenced LAB strain, *Streptococcus thermophilus* KLDS 3.1003 (GenBank Accession Number: CP016877) and its cell-free supernatant (CFS) can have high antimicrobial activity against food-borne and vaginal pathogens (*Staphylococcus aureus*, *Escherichia coli* and *Gardnerella vaginalis*) (unpublished data). Such exciting results can, among others, be integral in the development of useful ingredients in the development of novel functional foods, production of bio-drugs as well as bio-vaccines. The full characterization of antimicrobial substances produced by probiotic LABs is also becoming an important area of research as a result of their increasingly important industrial, nutritional and therapeutic applications. The main substances include exopolysaccharides (EPS), bacteriocins, biosurfactants and organic acids. Until now, most studies, for several limiting factors, have only been able to partially characterize these substances but this has to be improved on using new technologies so as to open new possibilities. Can probiotic LABs be used to develop anti-inflammatory yoghurts, cholesterol-lowering cheese, anti-diabetic ice creams, desserts and the likes as earlier postulated? Can interesting discoveries about the structure of LABs give new insights into their possible use in treating ailments of viral origin? Will more *in vivo* studies emerge, showing more direct correlations between the immuno-modulatory activities of LABs and their suppressing the onset of predisposing disease factors? The nearest future will hopefully answer these and many more.

Furthermore, research into the genetic components of probiotic lactic acid bacteria strains can also have tremendous advantages in eliminating incidences of food poisoning. Food poisoning currently accounts for an estimated 420,000 deaths/year which is deleterious to the economy of any nation [132]. This is rightly so as food is undoubtedly an important vehicle not only for nutrients but also for the favourable growth of pathogenic microorganisms. Given the growing interests in ensuring that foods consumed daily are both nutritious and safe [133,134], understanding how the genes of promising lactic acid bacteria strains suppress the gene expression and consequently the survival of reputed food-borne pathogens and their toxins may give new insights on the propensities of lactic acid bacteria as probiotics. Novel microencapsulation techniques for synbiotics should be optimized to ensure survival of more viable probiotic LAB cells for significant health impact [11,117,135]. This will be of particular importance to low- and middle-income countries which are most affected by food-borne diseases [130]. Bearing in mind that food safety is a shared responsibility, it must also be emphasized that consumer education be aggressively intensified to ensure that healthy food choices are constantly and consciously made as this is indispensable for healthy life and living. The nearest future may also see the emergence of more probiotic, prebiotic and symbiotic ice-cream products which may serve a variety of uses. The technological and non-technological drawbacks, some of which are highlighted in this review must first be tackled before desired significant advances can be made.

Based on the studies presented in this review, it is thus theoretically feasible that multiple strain combinations be used to investigate the attenuating effects of probiotic LABs and Bifidobacterium spp on gut microbiota with a view to reducing obesity and T2D. Similar studies comparing the effect of specific strains on the gut microbiota can be carried out with the view of developing new anti-obesity food and dairy products. Such studies may unveil new and novel pipelines for drugs and new food products with immense industrial applications. Some recent studies on the subject can serve as a strong theoretical platform for further researches into the modulation of the gut microbiota by testing the effects of single or multiple probiotic LAB strain dosages [15,44]. Panwar et al. [54] recently reported that although several Lactobacillus strains showed strong DPP-4 inhibitory activity, *Escherichia coli* 0157 and *Salmonella typhimurium* showed the strongest inhibition (30–32%). Investigations into the manipulation of these pathogenic microorganisms for probiotic purposes may thus be needful for future research. The nearest future may also see the emergence of novel starter cultures with more *in vivo* trials to support the claim that probiotic lactic acid bacteria may indeed have more direct effects on the suppression of pathways and processes within the human GIT that predisposes people to obesity and T2D. It is hypothesized that this line of research will greatly lower the current ethical, cultural or religious barriers impeding lactic acid biotechnology research and promotion of functional food ingredients.

## Conclusion

The application of probiotic LAB and research has received great attention for many years resulting in the development of various food products that possess diverse health benefits. This review has highlighted some of the areas where these beneficial bacterial strains have been applied, thus increasing the prospects of their use now and in the nearest future. Anti-obesity and anti-diabetic effects of LAB have been suggested in some of the studies reviewed. Their use as functional starter cultures in dairy products like yoghurt and ice cream show promising outcomes in the nearest future. Some more concerted *in vivo* studies may greatly lower current ethical, cultural and religious bottlenecks to probiotic lactic acid bacteria research. Key government and research institutes further investigate the mechanisms behind the attenuating effects of probiotic LABs to increase their applicability. Delivery techniques such as microencapsulation need further fine-tuning to optimize the survival of large numbers of viable probiotic LAB cells. Bioinformatics of probiotic LAB strains need further studies as well.
